# The Challenges Facing Training in Pediatric Surgery Worldwide

**DOI:** 10.3389/fped.2013.00024

**Published:** 2013-09-11

**Authors:** Spencer W. Beasley

**Affiliations:** ^1^University of Otago, Christchurch, New Zealand; ^2^Paediatric Surgery, Christchurch Hospital, Christchurch, New Zealand

**Keywords:** surgical training, surgical education, pediatric surgery, surgical workforce, neonatal surgery, international medical graduates, safe working hours, foreign aid

## Abstract

Like most specialties, pediatric surgery is becoming more complex, and changes to health systems have not always been in the best interests of trainees or their surgical teachers. This paper outlines four of the current challenges faced by training boards in pediatric surgery worldwide, and documents their implications for the future training of pediatric surgeons.

## Exposure to Rare and Complex Cases

While much of pediatric surgery involves the treatment of relatively simple but common surgical conditions, such as hernia and undescended testes, the most challenging conditions tend to be rare and are often quite complex, e.g., esophageal atresia or bladder exstrophy. It is their rarity and complexity that creates the challenge for training. Many pediatric surgeons, even at the completion of training, have had relatively little clinical exposure to these conditions. Paradoxically, it is in the emerging nations with large pediatric populations that may see the most cases, but they do not always have the trainees, or the well structured teaching programs and facilities for their treatment at the same level that is found in the better resourced health services. The situation is made worse by three additional factors: (1) the declining incidence of several of the structural congenital abnormalities of surgical significance; (2) changes to working hours and the failure of training boards in pediatric surgery to modify the configuration of their training programs to accommodate this; and (3) changes to the casemix of teaching hospitals as a result of funding imperatives.

### Changing incidence of congenital abnormalities of surgical significance

Improvements in nutrition and antenatal care, such as folate supplementation, have reduced the incidence of several of the major structural abnormalities, particularly neural tube defects. For example, women who become pregnant but have been taking a multivitamin with the B-vitamin folic acid prior to pregnancy have a 70% reduction in the risk of their baby having a neural tube defect (1, 2). One public health initiative has been to fortify bread with folate to ensure all women of child bearing age are so protected. Moreover, the accurate ultrasonographic diagnosis of spina bifida early in gestation has meant that many of these pregnancies are terminated, further reducing the incidence of live births of infants with spina bifida (this also applies to several other conditions with a poor prognosis).

There are several reports of a decline in the incidence of NEC (3) although this has not been observed in all institutions (4). Changes to the introduction of enteral feeding in VLBW infants as well as early recognition and prompt medical treatment reduce the likelihood of surgery and may diminish the trainee’s experience of this condition.

The incidence of anorectal malformations (ARMs) has marked regional variation. In first world countries the incidence is estimated to be about 2.0–2.5 per 10,000 live births, but is much higher in Africa and India. The reasons for the very wide variations in the prevalence between continents and countries may relate to nutritional factors and ethnicity. Despite limited data, there is an impression of an overall declining incidence, perhaps in part due to termination when it occurs in association with other more severe abnormalities, and partly to improvements in nutrition status prior to and during pregnancy. The end result is that many first world pediatric surgical trainees have relatively little exposure to these conditions.

Only the incidence of congenital abdominal wall defects, especially gastroschisis, seems to be on the increase, for reasons not clearly established. Recent data from the British Isles Network of Congenital Anomaly Registers (BINOCAR) confirm the increasing incidence of gastroschisis – from 2.5 per 10,000 total births in 1994 to 4.4 per 10,000 in 2004. Among babies of women aged under 20 the incidence of gastroschisis increased from 8.9 to 24.4 per 10,000 total births (5, 6). In New Zealand, the incidence of gastroschisis has increased from 2.96 to 5.16 per 10,000 live births between 1996 and 2004. Interestingly, during the same period, the incidence of exomphalos increased from 0.69 to 3.27 per 10,000 live births (7).

With the obvious exception of abdominal wall defects, the implication of these changes in incidence of structural congenital abnormalities is that trainees are exposed to fewer numbers of index neonatal cases, and this affects their training opportunities.

### Safe working hours and rostering changes

Changes to safe hours of practice, employment contracts, and to the configuration and rostering of surgical trainees has led to a diminution of both working hours and access to index cases. To date, the response to these changes by specialty training programs has been mostly of defiance and indignation, perhaps not recognizing that these changes are likely to be with us into the foreseeable future. It is with some urgency that training boards need to work out how to accommodate these restrictions through the use of new technology (e.g., simulation, web-based, and interactive teaching), restructuring of service/teaching commitments, development of better tools to assess competence and performance, and probably a faster move to competency-based training.

### Changing role of teaching hospitals affecting casemix

In Australasia, where training mainly occurs in public teaching hospitals, the casemix is changing as a result of resource and funding changes in the public sector. In general, this has meant constraints on the funding to the services provided by most of those institutions providing elective and acute pediatric surgical services. The public hospitals are increasingly being reserved for acute and complex care, with an increasing proportion of elective cases undergoing surgery in private hospitals, often out of reach of the trainee. There is an emphasis of efficiency of operative throughput and maximizing “productivity”; which means that trainees – whose involvement in surgery is often seen as slowing down operating lists and increasing operative time, and hence cost – have their training opportunities compromised. Although this drift in casemix mostly relates to the less complex cases it does affect the total operative exposure of the trainees.

As in other surgical specialties, simulation training in operative skills mainly has a role early on, at the time of acquisition of basic surgical skills. The likely contribution of simulation training in the latter stages of pediatric surgical training is less, where the emphasis is more focused on decision-making, clinical judgment, and other non-technical skills.

## Level of Supervision

Pediatric surgery still relies heavily on the apprenticeship model, albeit modified to a degree as training programs become more structured. A trainee works closely with one or more specialist surgeons for an extended period, and then moves to another training post where that same trainee is then exposed to, and learns from, another group of surgeons. Many programs require their trainees to move between hospitals, states, or even countries. In a training program that has a sound governance structure their progress is monitored closely by their specialty training board or equivalent, and this progress is reviewed at regular intervals. Many programs have a robust system of formative assessment. Most have some form of final exit examination that should focus on clinical and operative decision-making and professional judgment assessed at the correct cognitive level for someone completing specialty training (8).

There is a gradual trend away from time-based training to competency-based training, and this demands better recognition of prior learning, monitoring performance against pre-determined standards, and greater validation and reliability of the summative assessments.

All these changes demand a high level of supervision, measurement of performance, and provision of timely feedback – and this has been the great challenge for specialty training boards. The tools used remain rudimentary, variably applied, and even within the one training program their application often lacks consistency. There is a wide range of supervision provided to trainees according to the training post, with the level being more related to the inherent characteristics and culture of the training post than to the needs or seniority of the trainee (9). Specialty training boards claim that the information from in-training reviews that they receive from the surgical supervisors or surgical teachers is not always accurate; and sometimes it fails to identify the marginal or struggling trainee early on. Most training programs have some form of regular in-training formative assessment of progress that reports progress across a range of surgical competencies, but the quality and rigor of these assessments and the feedback they provide may vary significantly, which helps neither the training board nor the trainee. Indeed, the lack of consistency and reliability of regular formative assessment is considered the “Achilles heel” of many training programs: yet the measurement of trainee progress across all the areas of surgical competence is crucial for competency-based training.

In addition, the assessment of competence and performance has yet to become easily transferable between jurisdictions or countries. Most countries only recognize the specialty qualifications of a few other countries. Refinement and global acceptance of the metrics used to assess competence and performance is a pre-requisite for any consistency in training across borders and across countries (10).

## Training an Adequate Workforce

At present there appears to be a worldwide shortage of fully-trained pediatric surgeons. This shortage is most prominent in the developing nations, but exists intermittently or in some regions of many first world countries as well. It takes three forms:

(1) Contraction of senior staff appointments. In some countries, this is usually initiated at a governmental level but undertaken by local health bodies (e.g., hospitals, trusts, health boards, or through universities) ostensibly for budgetary reasons, to control health expenditure and health costs to the public sector; and
(2) Unfilled senior appointments. There may be a shortage of local applicants primarily because of a shortage of trainees completing training, or a mismatch between training capacity and available consultant positions. Workforce predictions invariably underestimate the number of trainees required to sustain a service to meet community needs, and this puts some pressure on these jurisdictions to seek applicants from elsewhere (which generally means surgeons from overseas – *vide infra*). The process for foreign doctors is often not easy: applicants from overseas (International medical graduates, IMGs) may find it difficult to meet the new jurisdiction’s requirements and encounter many impediments. These range from those put in place by the registering authorities, such as medical councils and medical boards, to those placed by the relevant specialty association concerned that the IMG should be able to demonstrate comparability in their training programs and performance. This may include complete “retraining,” to the requirement to pass the final summative examinations or to a period of supervision or assessment.
(3) In less developed countries the main impediment is a lack of an existing specialty service or the resources to run a quality training program in all specialties. This applies to pediatric surgery as much as or more than any other surgical specialty because it is relatively small, even though ironically these countries often have a very high pediatric population as a percentage of total population compared with first world countries. To make matters worse, they have high surgical needs due to the additional diseases to which children and adolescents are exposed to as a result of climate, infections, poor sanitation, poverty, poor primary health infrastructure, and delayed access to surgical services.

A crude indicator of the shortfall in pediatric surgical workforce is the percentage of children not having their elective surgery performed by a pediatric surgeon. Yet the greatest benefit from a regional pediatric surgery service can be for minor elective cases, such as for hernias and undescended testes (11). The disparity in outcomes for infants with pyloric stenosis (12) (CAA) and intussusception (13), according to whether they are managed by a specialist pediatric surgical unit, is well established.

Other challenges faced by those governing pediatric surgical training which have the potential to affect the future workforce and its configuration include the high percentage of women in the specialty (14), the desire for part-time or interrupted training, and subspecialization. There would be few centers where even those who have largely limited their elective work to a narrow scope within pediatric surgery would not still be required to contribute to the acute service provision.

## Support to Emerging Countries

There are huge inequities in the percentage of GDP (Gross Domestic Product) devoted to health, and even larger differences in the dollar amounts provided to health services. Data on this disproportionate resourcing of health services worldwide are freely available (15). In the poorer countries, most specialties are affected, with pediatric surgery being no exception.

The problems affecting the poorer and emerging nations extend well beyond resourcing of services and the facilities and equipment that they require. There may be either no or only a rudimentary training program. Indeed, the country may have neither the population nor the resources to have its own formal training program. As a consequence, the training of pediatric surgeons often requires a period working overseas in a specialized pediatric surgical unit. Less well resourced countries may be further handicapped by having only a few pediatric surgeons in total, and have limited equipment, support services (especially pediatric-trained anesthetists), or capacity locally. The dilemma is that it is those local trainees with the greatest potential, the “best brains,” are forced to gain at least some of their experience overseas; and because they are so talented, and because of the lifestyle advantages that may be offered, they are tempted or encouraged to stay overseas. Often they gain (and are highly successful) in senior roles in highly regarded institutions, but their original community misses out. At some point well resourced first world countries will need to address this issue, from both an ethical and moral standpoint.

Historically, surgeons from developed countries have taken pride in their (usually) *pro bono* and voluntary assistance to less privileged countries. Typically they travel to a so-called “third world” country as part of a surgical team and operate there frantically for a week or two. It is true that some individual patients may benefit – provided they get no major complications after the team has departed – but overall the capability and capacity of the local service has not been enhanced. Sometimes these visits even compromise the reputation and standing of the local surgeons in the eyes of their community – which is not to their long term benefit (“look what the clever overseas surgeons can do: they are so much better than our own”). Other forms of “assistance” include sending relatively untrained and junior students or graduates for an elective period to an under-resourced country: this is often done through a sponsoring university. The danger here is that arguably most of the benefit is derived by the donor organization and to the visiting individual students rather than to the recipient nation where the help may be of limited quality and leaves no long term gain in expertise locally. It is my belief that all assistance of this type must respect the local needs and ensure that the capability and infrastructure of the recipient country is improved.

There are several ways in which this can be done. Perhaps the most important is to identify early on young surgeons (even medical students) who have the potential to be future leaders of surgery in their country, and then to support and nurture them through surgical training (often with a period overseas) until they are at a standard where they can return to their homeland. Training should be fashioned to align with local needs. This can only be done with a genuine longer term commitment which is sensitive to local aspirations. There is an expectation that many of these talented individuals ultimately will be the leaders in the development of their specialty service regionally – it is a long term investment.

One of the impediments preventing these surgeons from returning to their native land is the lack of an appropriate and resourced job to go to. Often they receive quality training overseas but then find there is no political willingness (seen as the burden of another salary) or capacity for them to return home to a position that is supported and viable. This places an obligation on those sponsoring or organizing their training overseas to engage at a high political or ministry of health level to ensure there is available employment at home once they have completed their overseas training. Also, the necessary linked services (in the case of pediatric surgery this typically includes nursing, pediatric anesthetic, pathology, and pediatric imaging services) must be sufficiently developed to allow the pediatric surgical service to be sustainable. Help from privileged countries, no matter how well intentioned, is only of lasting value if it improves the infrastructure and sustained capability of the local service.

This paper has outlined some of the challenges facing pediatric surgery worldwide. One would hope that when reviewed in 10 years that we could look back and say we had made significant progress!

## Conflict of Interest Statement

The author declares that the research was conducted in the absence of any commercial or financial relationships that could be construed as a potential conflict of interest.
